# Inline balloon-assisted vascular sheath fragment removal

**DOI:** 10.1186/s42155-020-00142-1

**Published:** 2020-10-04

**Authors:** Neema Jamshidi, Jason Chiang

**Affiliations:** Department of Radiological Sciences, UCLA, 757 Westwood Ave, Ste 2125, Los Angeles, CA 90095 USA

**Keywords:** Foreign body retrieval, Intravascular, Intra-arterial

## Abstract

**Background:**

Unretrievable foreign bodies are associated with high morbidity and mortality. While the majority of reported cases involve the venous circulation, intra-arterial foreign body displacement have the potential to migrate more distally with a higher risk for dissection and hemorrhagic complications during retrieval. As the number of intravascular procedures continues to increase, there is also likely to be a concomittant increase in the number of retrieval procedures, particular for fractured catheters and sheaths. Although snaring is frequently the traditional, ‘go-to’ method for retrieval, there are inherent risks of further dislodgement or fracture.

**Case report:**

We describe a case that involves retrieval of a fractured sheath that originated in the common femoral artery but migrated into the popliteal artery. Different retrieval approaches were employed, however ultimately balloon assisted, over-the-wire retrieval was the successful approach.

**Conclusions:**

We anticipate that over-the-wire, inline-retrieval approaches will continue to grow in popularity and use, particularly with respect to manipulation within the arterial circulation.

## Background

Intravascular foreign body dislodgement and embolization is a potential complication of any percutaneous image-guided procedure. While loop snaring is frequently employed for foreign object retrieval, this technique is suboptimal when the foreign object does not have traction or is at risk for fragmentation. In this case presentation we describe retrieval of a fragmented vascular sheath that migrated into the peripheral arterial system. A balloon catheter was negotiated distal to the fragmented vascular sheath and then partially inflated the balloon. Ultimately, due to the orientation of the fragmented vascular sheath, it was possible to thread it and safely retrieve the vascular sheath.

## Case presentation

**Fig. 1:**
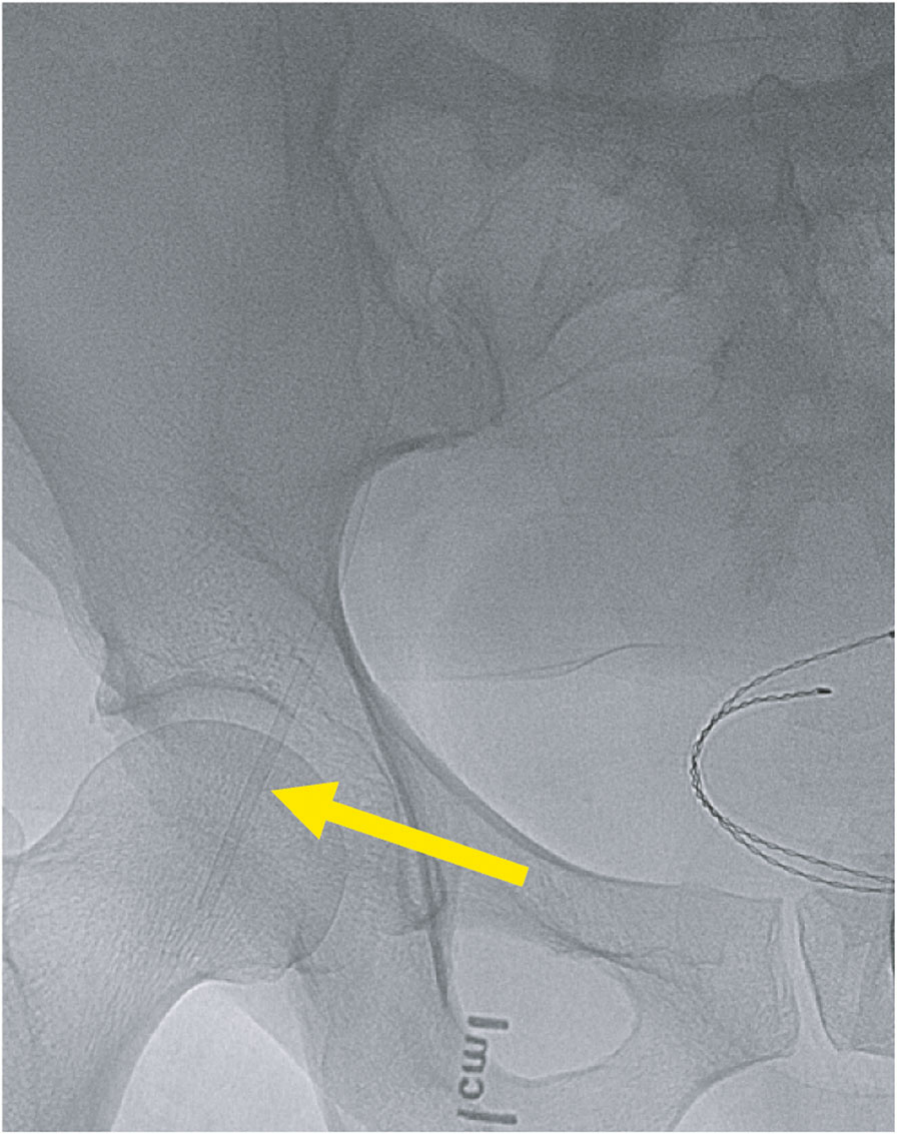
Initial fluoroscopic image showing the sheath fragment trapped within the right common femoral artery (yellow arrow)

**Fig. 2:**
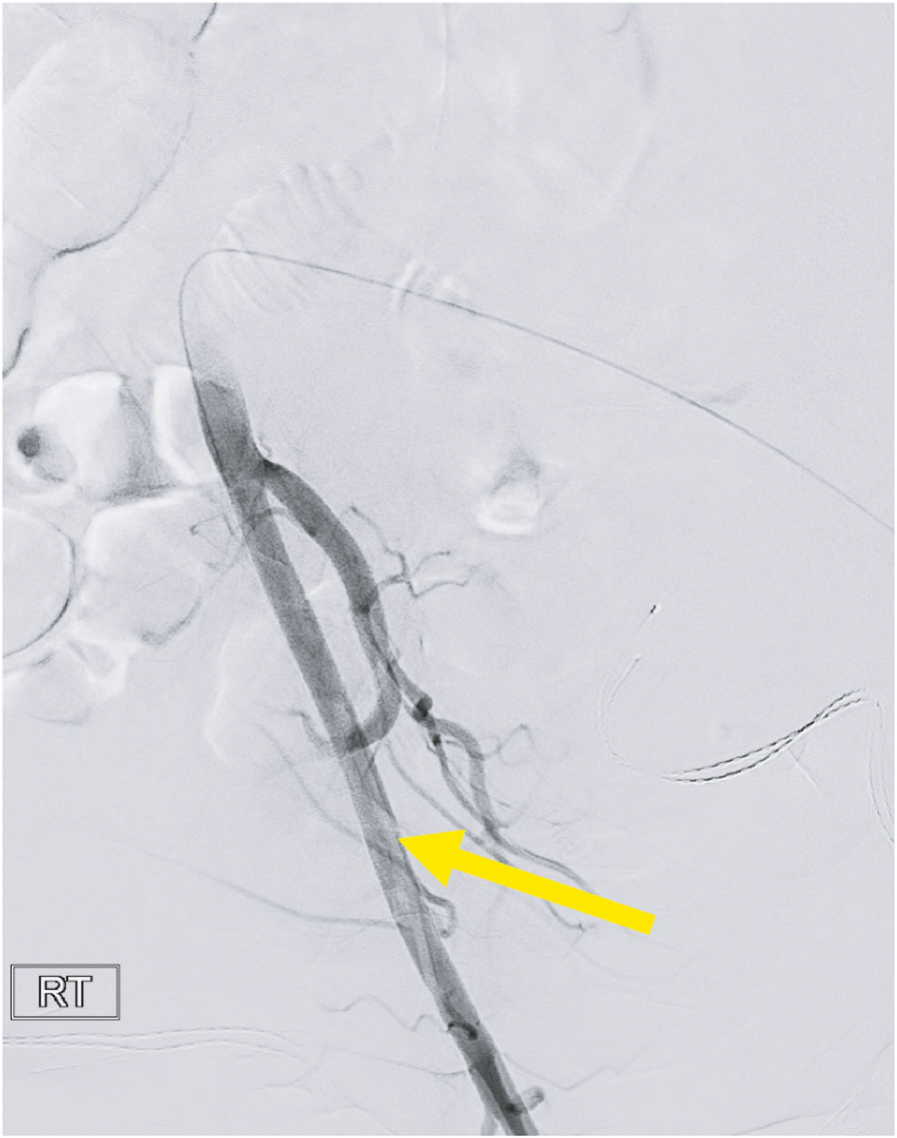
Snapshot from digital subtraction angiogram (DSA), clearly demonstrating intravascular position of the sheath

A patient undergoing a cerebral angiogram was found to have a malfunctioning right common femoral artery (CFA) 6 French sheath. The sheath could not be aspirated and the tip was inadvertently separated from the hemostatic valve-housing at which point interventional radiology was consulted for retrieval. Initial fluoroscopy and angiography revealed the dislodged sheath tip to be located at the level of the femoral head without evidence of thrombus or occlusion (Figs. 1 and 2). No skin hematoma or external protrusion of the sheath was visualized, and the dislodged sheath tip was confirmed to be entirely intravascular.

**Fig. 3:**
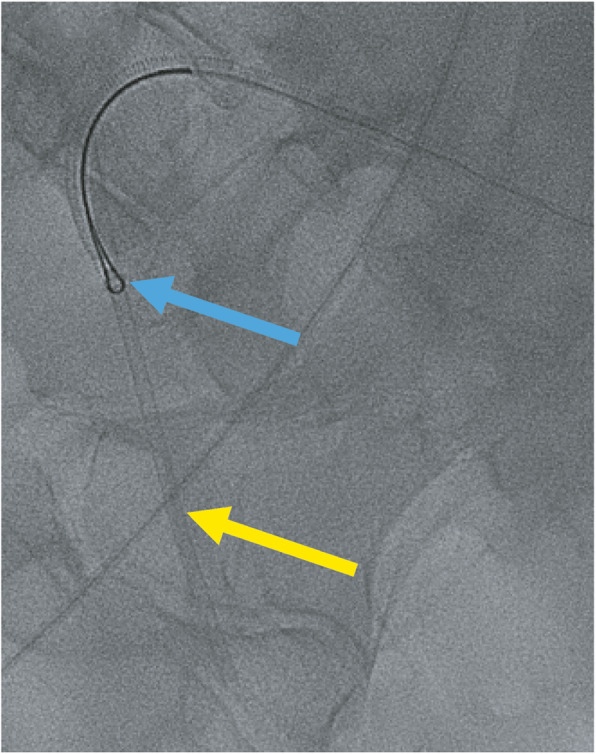
Initial Gooseneck snaring of the catheter fragment (blue arrow) and retraction of the sheath fragment (yellow arrow)

**Fig. 4:**
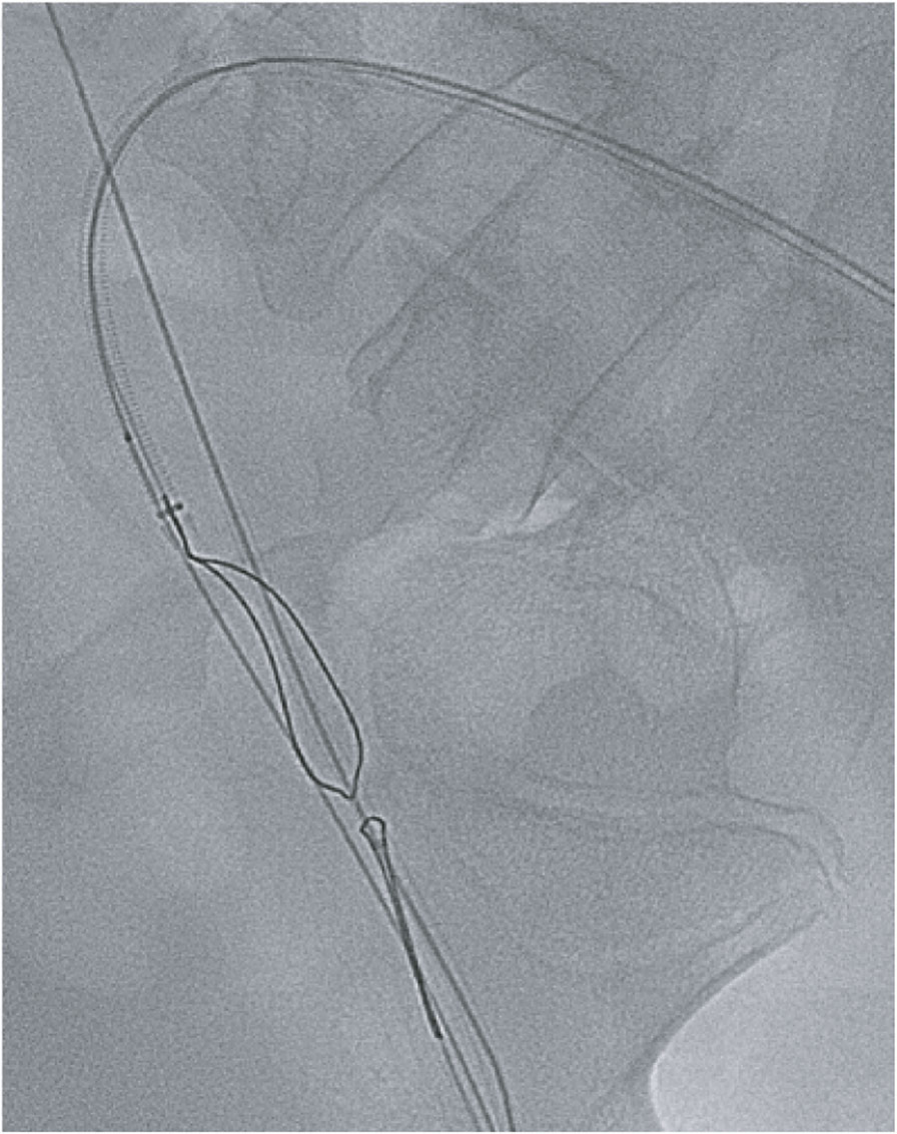
Snaring of the foreign body from both ends and attempted retraction in the Destination sheath that had been advanced from the contralateral side

An 8 French × 45 cm Destination Guiding Sheath (Terumo Medical Corporation, Somerset, NJ) was advanced up and over from the left CFA into the right external iliac artery. A 25 mm Amplatz Goose Neck Snare (Medtronic, Minneapolis, MN) was used to secure the dislodged sheath tip (Fig. 3), but could not retract the catheter fragment into the sheath. Upon further manipulation, the dislodged sheath tip migrated past the right popliteal artery and into the tibioperoneal trunk (Fig. 4).

**Fig. 5:**
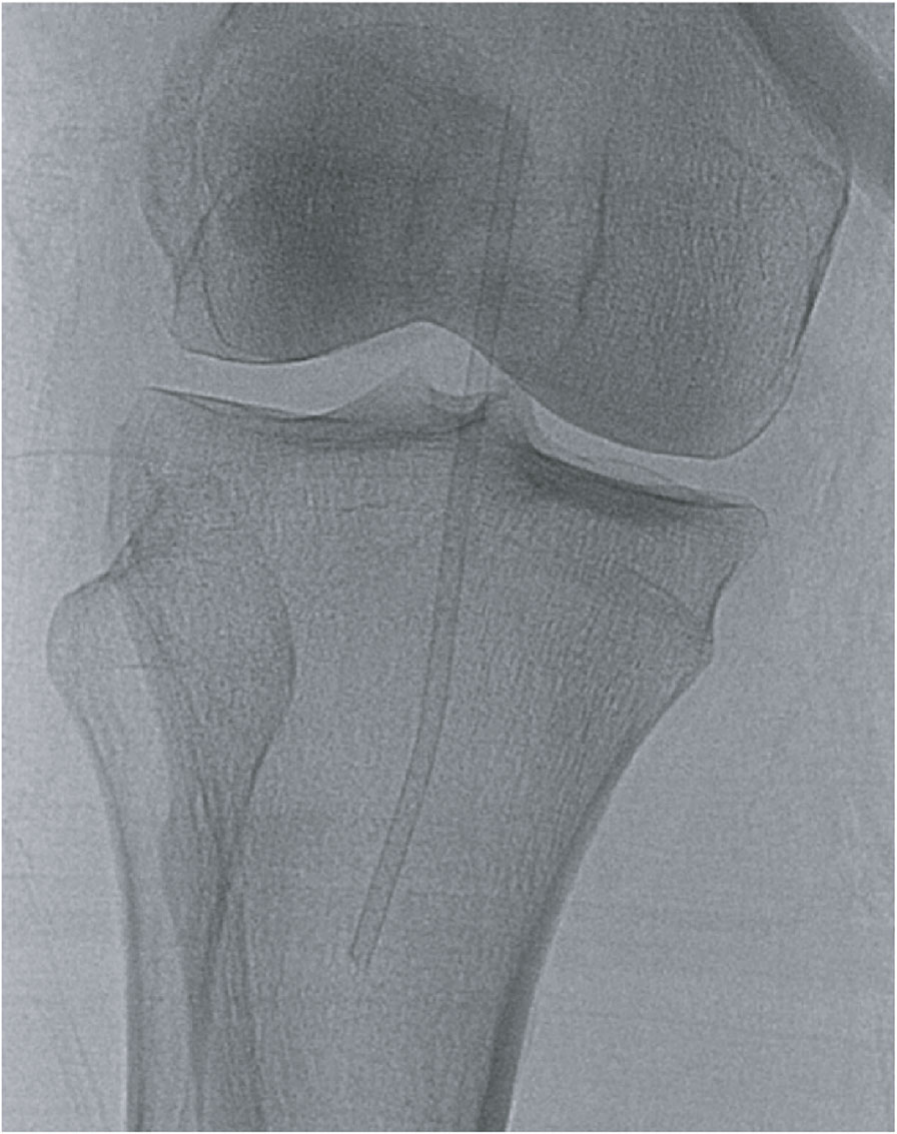
Fluoroscopic image showing the migrated position of the sheath fragment extending from the popliteal artery into the tibioperoneal trunk

**Fig. 6:**
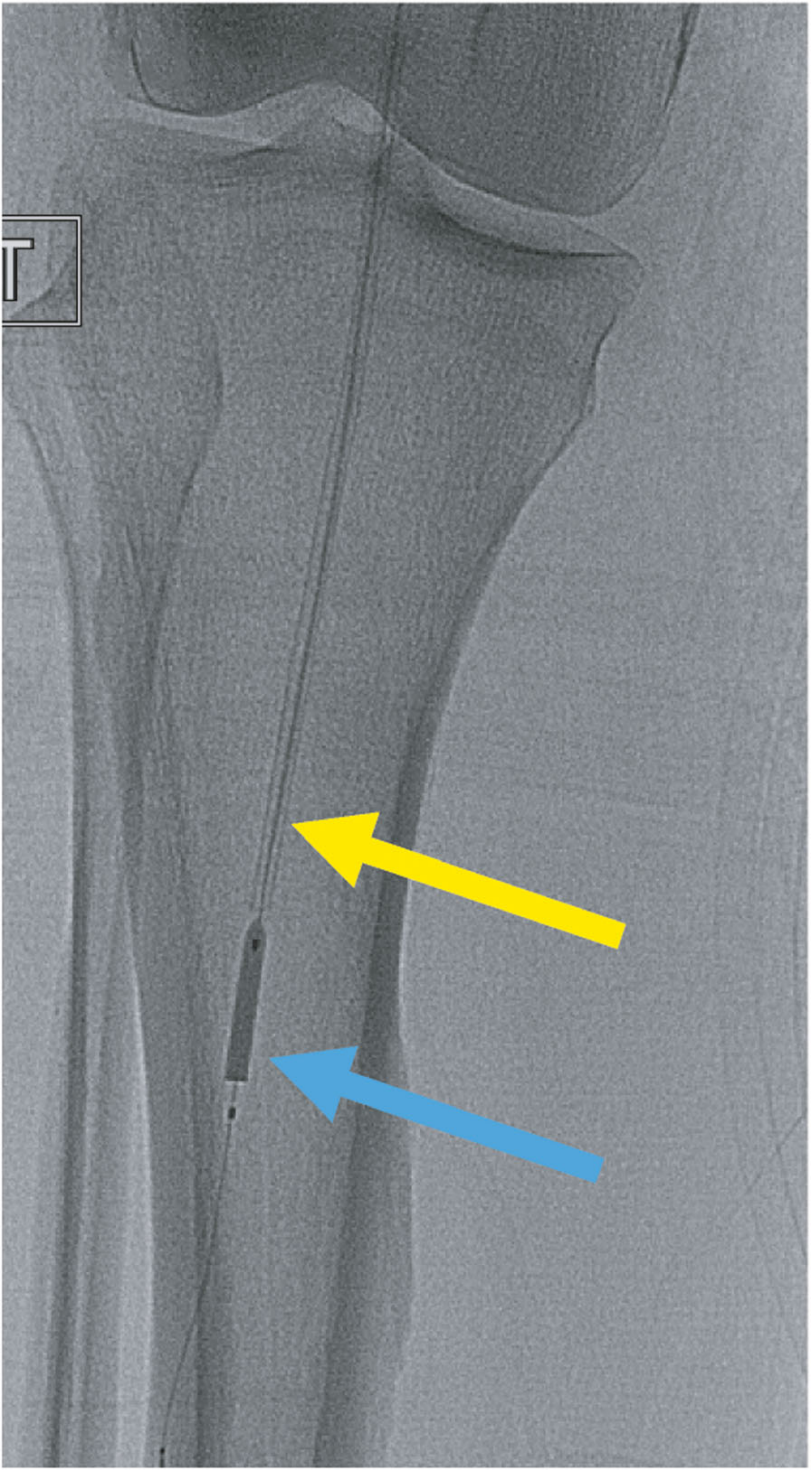
Cannulation and capture of the catheter fragment (yellow arrow) using a partially inflated Sterling balloon past the end of the sheath fragment (blue arrow)

**Fig. 7:**
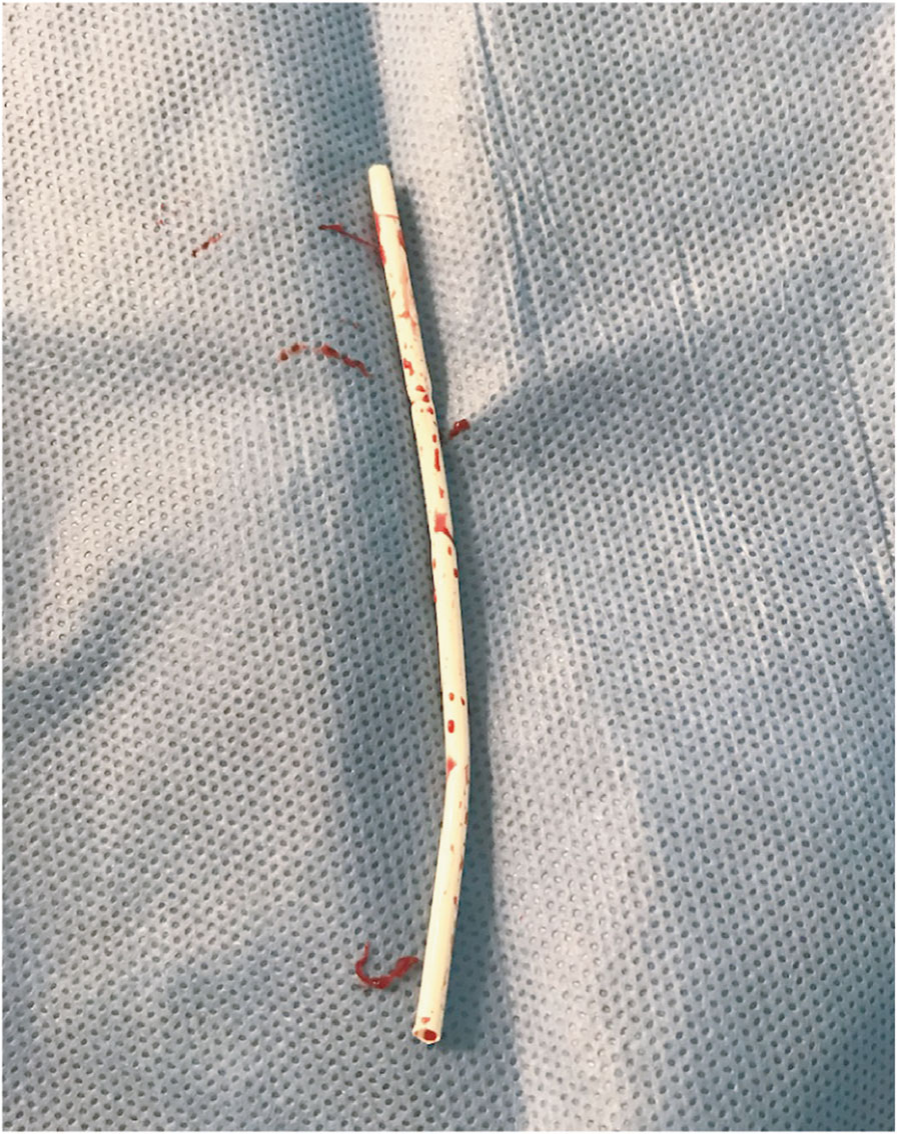
Image of the extracted sheath fragment

At this point the retrieval strategy was changed and the dislodged sheath tip was cannulated with a 0.014″ Hi-Torque Balance Middleweight (BMW) guidewire (Abbott Vascular, Santa Clara, CA), enabling advancement of a 3 mm × 4 cm Sterling balloon (Boston Scientific, Marlborough, MA) past the distal end of the dislodged sheath tip (Fig. 5). The Sterling balloon was partially inflated and then retracted, trapping the dislodged sheath tip against the Destination sheath. Visualized inspection and spot film confirmed complete removal of the dislodged sheath tip (Fig. 6) following removal of the sheath (Fig. 7).

## Discussion

Intravascular foreign bodies that cannot be retrieved are associated with a high morbidity and mortality rate, reaching rates as high as 71% (Fisher and Ferreyro 1978). Most reported cases of broken catheter retrievals involve the venous circulation, where larger fragments may migrate into the right ventricle and pulmonary circulation. Intra-arterial cases, on the other hand, migrate more distally as a result of blood flow and are at a higher risk for bleeding and embolization during retrieval. Despite the high incidence of broken catheters within arterial vasculature, the retrieval of these foreign objects is rarely reported (Ramachandran et al. 2016; Gupta et al. 2005). Turning to surgical options are associated with high morbidity as well, especially if patients are critically ill and require less invasive approaches (Hehir et al. 1992).

Percutaneous removal provides a variety of options and is considered safer with less morbidity. Various techniques are available for percutaneous removal of intravascular foreign bodies such as loop snare, proximal and distal grab technique, coaxial snare technique, lateral grasp technique, dormie baskets, and small balloon catheter technique. Of these various techniques, the loop snare method is most frequently employed (Egglin et al. 1995; Carroll et al. 2013). Loop snares are widely available and have the advantage of being flexible enough to follow a variety of curvatures related to vascular anatomy (Tytle et al. 1995; Koseoglu et al. 2004). The inception of nitinol-based loop snares have added additional flexibility as they are able to maintain their shape within the blood vessels. However, loop snaring is limited by their variable gripping ability and can be of limited utility if the free ends of the foreign body are not available to snare. Out of the reported failures of the catheter snaring strategy, getting the fragment ends onto the same plane as the snare is a commonly cited difficulty (Gupta et al. 2005; Rossi 1970).

The balloon-assisted strategy has certain advantages that should be highlighted, particularly when working within the arterial system. With small, more delicate foreign objects, there is always the concern that forceful pulling, either via snaring or blunt pulling, will cause further fragmentation, which can account for up to 60% of endovascular loss during retrieval (Carroll et al. 2013). A prior study demonstrated 40% of patients with arterial foreign bodies using snare technique suffered occlusive arterial spasms or repositioning to another vessel that was amenable to surgical cutdown (Egglin et al. 1995). The balloon assembly would minimize the risk for further comorbidities by not localizing the traction to any particular point on the catheter or vascular wall. The balloon technique described here has previously been utilized for retrieving lost stents, primarily in the setting of interventional cardiology procedures (Gupta et al. 2005; Karaca et al. 2016; Mehta et al. 2014). In a prior study, only 2/24 (8.3%) endovascular retrievals were performed using balloon catheters, both of which were used to retrieve broken catheter sheaths. Out of these two procedures, only one was successfully performed, while the other was left in place (Carroll et al. 2013). The benefits of inline balloon retraction of foreign bodies over snare-based techniques have been described by Gupta et al. (Gupta et al. 2005). The technique described here builds upon that principle by advancing the ballon past the distal end of the catheter fragment; this allows 1) more secure capture of the fragment, 2) requires less inflation of the balloon, and 3) minimizes potential trauma to the vascular endotherium and intima.

## Conclusion

With the continued growth in the number of percutaneous procedures performed, there is an expected concomitant increase in foreign object retrieval cases. Since many foreign objects often have cylindrical cross-sectional profiles, the approach described herein provides a safe and secure method of retrieving objects while minimizing intimal trauma. Although snaring is the traditional method for retrieval, there are inherent risks of further dislodgement or fracture. The aforementioned technique for retrieval utilizing a partially-expanded balloon to safely secure and remove foreign bodies within the arterial system is readly available and easily implemented. We anticipate that over-the-wire, inline-retrieval approaches will continue to grow in popularity and use, particularly with respect to manipulation within the arterial circulation.

## Data Availability

Additional data (DSA runs, etc) is available in de-identified format upon request from the authors.

## Abbreviations

BMW: Balance Middleweight Wire
CFA: Common femoral artery
DSA: Digital subtraction angiogram

## References

[CR1] Carroll MI, Ahanchi SS, Kim JH, Panneton JM (2013). Endovascular foreign body retrieval. J Vasc Surg.

[CR2] Egglin TK, Dickey KW, Rosenblatt M, Pollak JS (1995). Retrieval of intravascular foreign bodies: experience in 32 cases. AJR Am J Roentgenol.

[CR3] Fisher RG, Ferreyro R (1978). Evaluation of current techniques for nonsurgical removal of intravascular iatrogenic foreign bodies. AJR Am J Roentgenol.

[CR4] Gupta AK, Purkayastha S, Krishnamoorthy T (2005). Percutaneous retrieval of intravascular broken catheter fragments. A novel technique using a balloon. Interv Neuroradiol.

[CR5] Hehir DJ, Cross KS, Kirkham R, Moore DJ, Shanik DG (1992). Foreign body complications of central venous catheterisation in critically ill patients. Ir J Med Sci.

[CR6] Karaca O, Cakal B, Omaygenc O, Turkmen M (2016). Percutaneous retrieval of an embolized catheter tip with the balloon dilatation technique. Res Cardiovasc Med.

[CR7] Koseoglu K, Parildar M, Oran I, Memis A (2004). Retrieval of intravascular foreign bodies with goose neck snare. Eur J Radiol.

[CR8] Mehta V, Pandit BN, Yusuf J, Mukhopadhyay S, Trehan V, Tyagi S (2014). Retrieval of impacted broken balloon by balloon inflation in guiding catheter. Cardiovasc Interv Ther.

[CR9] Ramachandran P, Reddy RP, Rao MS, Jayaram AA (2016). A novel approach for the retrieval of broken catheter fragment - using balloon dilatation technique. J Clin Diagn Res.

[CR10] Rossi P (1970). “Hook catheter,” technique for transfemoral removal of foreign body from right side of the heart. Am J Roentgenol Radium Therapy, Nucl Med.

[CR11] Tytle TL, Prati RC, McCormack ST (1995). The “gooseneck” concept in microvascular retrieval. AJNR Am J Neuroradiol.

